# Sample deposition onto cryo-EM grids: from sprays to jets and back

**DOI:** 10.1107/S2059798320002958

**Published:** 2020-03-25

**Authors:** David P. Klebl, Diana C. F. Monteiro, Dimitrios Kontziampasis, Florian Kopf, Frank Sobott, Howard D. White, Martin Trebbin, Stephen P. Muench

**Affiliations:** a School of Biomedical Sciences, University of Leeds, Leeds LS2 9JT, United Kingdom; b Astbury Centre for Structural and Molecular Biology, University of Leeds, Leeds LS2 9JT, United Kingdom; c The Hamburg Centre for Ultrafast Imaging, Universität Hamburg, Hamburg, Germany; d Hauptman–Woodward Medical Research Institute, Buffalo, New York, USA; e Institute of Business, Industry and Leadership, University of Cumbria, Carlisle CA1 2HH, United Kingdom; f School of Molecular and Cellular Biology, University of Leeds, Leeds LS2 9JT, United Kingdom; g Department of Chemistry, Biomolecular and Analytical Mass Spectrometry Group, University of Antwerp, Antwerp, Belgium; h Department of Physiological Sciences, Eastern Virginia Medical School, Norfolk, Virginia, USA; i Department of Chemistry, State University of New York at Buffalo, New York, USA

**Keywords:** time-resolved, cryo-EM, sample preparation, gas dynamic virtual nozzle, structure determination, microfluidics

## Abstract

Despite the great strides made in the field of single-particle cryogenic electron microscopy (cryo-EM) in microscope design, direct electron detectors and new processing suites, the area of sample preparation is still far from ideal. Traditionally, sample preparation involves blotting, which has been used to achieve high resolution, particularly for well behaved samples such as apoferritin. However, this approach is flawed since the blotting process can have adverse effects on some proteins and protein complexes, and the long blot time increases exposure to the damaging air–water interface. To overcome these problems, new blotless approaches have been designed for the direct deposition of the sample on the grid. Here, different methods of producing droplets for sample deposition are compared. Using gas dynamic virtual nozzles, small and high-velocity droplets were deposited on cryo-EM grids, which spread sufficiently for high-resolution cryo-EM imaging. For those wishing to pursue a similar approach, an overview is given of the current use of spray technology for cryo-EM grid preparation and areas for enhancement are pointed out. It is further shown how the broad aspects of sprayer design and operation conditions can be utilized to improve grid quality reproducibly.

## Abstract 2

Sample preparation within single-particle cryo-electron microscopy can still be a significant bottleneck, with issues in reproducibility, ice quality and sample loss. New approaches have recently been reported that use spraying or pin printing instead of the traditional blotting approach. Here, experience in the use of different nozzle designs and spraying regimes is reported together with their influence on the resulting spray and grid quality.

## 1. Introduction   

Over the last decade, cryo-electron microscopy (cryo-EM) has emerged as a major technique for the high-resolution structure determination of proteins and protein complexes (Callaway, 2015 ▸; Cheng, 2018 ▸). However, sample preparation is regarded as one of the main bottlenecks in cryo-EM, and research into novel grid-preparation methods has gained much attention. The traditional blotting technique was introduced in the 1980s by Dubochet and coworkers (Dubochet *et al.*, 1985 ▸). In the blotting approach, typically 3 µl of protein solution is applied to a cryo-EM grid, with subsequent blotting leaving only a thin liquid film on the grid (≤100–200 nm thick). The thin film is then vitrified, typically by plunging into liquid ethane, and can be transferred for imaging in the electron microscope. While the blotting method has undoubtedly been very successful in producing high-resolution sub-2 Å resolution structures (Zivanov *et al.*, 2018 ▸), its widespread use, and the increasing popularity of cryo-EM in general, have revealed its shortcomings. A large amount of sample is wasted through blotting, with 99.9% of the sample being removed by the blotting paper (Arnold *et al.*, 2017 ▸). The interactions between the filter paper and the sample are not yet fully understood, but a recent study suggests that the process is much less controlled than one might expect (Armstrong *et al.*, 2019 ▸). In addition, contaminants such as divalent cations may leach from the filter paper, which can be detrimental to the sample (Walker *et al.*, 1994 ▸). The blotting step is typically conducted in between 2 and 10 s, a period of time that allows the sample to adhere to surfaces such as the air–water interface, adopt a preferred orientation or even denature, which can hinder high-resolution structure determination (D’Imprima *et al.*, 2019 ▸; Noble *et al.*, 2018 ▸). Moreover, this (relatively) long time of blotting does not allow the trapping of reaction intermediates on the millisecond timescale.

Consequently, a whole new generation of freezing tech­niques is currently under development. Many of these techniques apply much lower sample volumes directly onto the grid. There are well understood ways to generate small liquid droplets that were developed for various applications such as combustion, drug inhalation or surface coating (Gañán-Calvo, 1998 ▸). The cryo-EM field has turned to these approaches and reported their use for the preparation of cryo-EM grids. The Spotiton system employs piezo-electric dispensers (such as those used in inkjet printers) to deliver small amounts of liquid in a highly controlled manner (Jain *et al.*, 2012 ▸; Dandey *et al.*, 2018 ▸). The VitroJet uses pin-printing technology to remove blotting and reduce sample volume to the nanolitre scale (Ravelli *et al.*, 2019 ▸). The Frank group has used direct-pressure atomizers for rapid sample delivery onto cryo-EM grids, which allowed capture of the ribosome in transient conformational states at near-atomic resolution (Kaledhonkar *et al.*, 2019 ▸). The use of ultrasonic nozzles for spraying has been independently described by two groups and has proven to be capable of producing grids for high-resolution structure determination (Ashtiani *et al.*, 2018 ▸; Rubinstein *et al.*, 2019 ▸).

However, the major prerequisite for cryo-EM imaging, and a significant challenge when spraying a sample, is the thinning of the droplets on the grid to ≤100–200 nm prior to vitrification. Droplet thinning is a function of the surface properties of the grid, as discussed by Jain *et al.* (2012 ▸). The success of self-wicking grids for Spotiton (Wei *et al.*, 2018 ▸) and Shake-it-off (Rubinstein *et al.*, 2019 ▸) demonstrates the importance of the grid surface properties for thinning. In contrast, gas-assisted and ultrasonic sprays can also produce thin liquid films using regular grid types. Therefore, spray characteristics also play an important role in droplet thinning on grids, alongside the surface properties of the grid. This is further underpinned by work from Frank and coworkers who, in their spraying approach, show a correlation between droplet size and ice thickness (Feng *et al.*, 2017 ▸). However, to date there are no publications that describe the spray behaviour for these devices in detail, which limits the development of this approach by other groups.

We have previously reported the use of another variation of cryo-EM grid preparation, voltage-assisted spraying, to enable rapid mixing and freezing for time-resolved cryo-EM studies (TrEM; Kontziampasis *et al.*, 2019 ▸). In the voltage-assisted spraying approach, the sample is applied onto a fast-moving (≤3 m s^−1^) cryo-EM grid. Between the droplets landing on the grid and freezing in liquid ethane, the liquid forms a thin film, allowing high-resolution imaging. In this work, we set out to deepen our understanding of the parameters that control droplet spreading when using standard cryo-EM grids. We hypothesize that droplet size and droplet speed play important roles in sufficiently thinning the droplets prior to freezing. Based on these findings, we compare different methods for depositing droplets with different sizes and speeds onto EM grids to produce grids for biological structure determination by cryo-EM.

## 2. Methods   

Cryo-EM grid-preparation experiments were performed using the previously described setup for voltage-assisted spraying (Kontziampasis *et al.*, 2019 ▸). The only significant modification to the setup was the new nozzles, which are described below. Quantifoil 200 mesh Cu R2/1 or 300 mesh Cu R1.2/1.3 grids were glow-discharged in air for 99 s at 10 mA and 0.1 mbar using a Cressington 208 carbon coater with a glow-discharge unit and were used within 30 min after glow discharge. No voltage was applied to the liquid/nozzle when preparing grids using capillaries for Rayleigh jets or gas dynamic virtual nozzles (GDVNs).

Analysis of the voltage-assisted spraying approach was conducted on the previously presented grids of equine apoferritin, *Escherichia coli* ribosome and porcine thin filaments (Kontziampasis *et al.*, 2019 ▸). To estimate the relation between droplet diameter in-flight and on-grid the contact-angle estimation of Jain *et al.* (2012 ▸) was used (θ = 10–15°). The height and radius of the spread droplet were estimated using the following equations (Jain *et al.*, 2012 ▸),
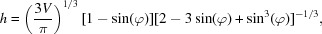


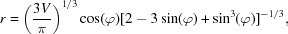
where φ = (π/2) − θ, *V* is the droplet volume, *h* is the height and *r* is the radius of the spread droplet. We note that while this model seems to hold true for large droplets from voltage-assisted spray and Rayleigh jets, it cannot explain the larger spread areas of thin ice produced by droplets from GDVNs or by the smaller droplets in the voltage-assisted approach.

For all grids prepared using Rayleigh jets, apoferritin from equine spleen (Sigma–Aldrich, catalogue No. A3660) was used as a test sample at 10 µ*M* (24-mer) in 30 m*M* HEPES, 150 m*M* NaCl pH 7.5. For the generation of Rayleigh jets, either a 10 or 50 µm internal diameter (ID) capillary was used (PicoTip SilicaTip emitter with 10 µm ID or TaperTip emitter with 50 µm ID). The capillaries were connected to the computer-controlled syringe pumps using fluorinated ethylene propylene (FEP) tubing with appropriate IDs (Upchurch, 1/16′′ outer diameter). Grid speeds were between 0.7 and 1.4 m s^−1^, the capillary tip was positioned 7 mm from the grid trajectory and the vertical distance between the capillary and liquid ethane was 3 cm. The liquid flow rates were 1 and 8 µl s^−1^ for the 10 and 50 µm capillaries, respectively.

Microfluidic GDVN devices were produced as described previously (Trebbin *et al.*, 2014 ▸). In short, the liquid-jet geometries were designed in *AutoCAD* (Autodesk) in a three-layer design. The first layer determined the main fluid inlet and the nozzle gas-flow focusing geometry, the second layer introduced the 3D gas-flow focusing with gas. The third layer contained the remaining necessary structural features for correct alignment during fabrication. The nozzle parameters are listed in Supplementary Table S1.

The *AutoCAD* structures were transferred to a chromium photolithographic mask (MB Whitaker, 4 × 4 × 0.09′′ soda lime glass coated with AZ1518 photoresist) using laser lithography. The designs were transferred to a layered SU8-3025 (negative photoresist, Michrochem) using UV photolitho­graphy with an MJB4 mask aligner (SUSS Microtec). The photoresist was spin-coated to the desired layer heights and baked, exposed and developed following the manufacturers’ guidelines. These steps were repeated to build a multi-layered structure. After development, the SU8 master was used to produce the microfluidic nozzles using soft lithography techniques using polydimethylsiloxane (PDMS) as described previously (Trebbin *et al.*, 2014 ▸). 10:1 PDMS:curing agent mixtures were used for fabrication of the final GDVN devices.

High-speed imaging of the jets/sprays was performed using a Photron SA-Z at a recording rate of 200 000 frames per second (fps). While the electronic shutter time was 159 ns, the actual exposure of the frame was determined by the 10 ns pulses of the illumination laser (640 nm; Cavilux Smart UHS, Cavitar). The high-speed camera and illumination laser were connected to an IX73 microscope (Olympus). The flow tests were performed using neMESYS high-precision syringe pumps (Cetoni).

Grids for cryo-EM data collection were prepared using 20 µ*M* apoferritin (Sigma–Aldrich, catalogue No. A3660) in 30 m*M* HEPES, 150 m*M* NaCl pH 7.5. The grid speed was 0.7 m s^−1^, the capillary tip was positioned 7 mm from the grid trajectory and the vertical distance between the capillary and liquid ethane was 2.5 cm, resulting in a time delay of 36 ms between spraying and freezing.

All cryo-EM was performed on FEI Titan Krios microscopes at the Astbury Biostructure Laboratory in Leeds. The apoferritin data set was collected on Titan Krios 2 equipped with a Gatan Bioquantum energy filter (20 eV slit) and a Gatan K2 Summit direct electron detector operated in counting mode. Briefly, 690 micrographs were collected and corrected for beam-induced motion with *MotionCor*2 (Zheng *et al.*, 2017 ▸), and the contrast transfer function was estimated using *Gctf* (Zhang, 2016 ▸) in *RELION*-3 (Zivanov *et al.*, 2018 ▸). Particles were picked with *crYOLO* 1.3 using the general model (Wagner *et al.*, 2019 ▸). All further processing was performed in *RELION*-3. After one round of 2D and 3D classification, good particles were taken forward to refinement (with octahedral symmetry), Bayesian polishing (Zivanov *et al.*, 2019 ▸), per-particle CTF and beam-tilt estimation. The final resolution was 3.5 Å according to the FSC = 0.143 criterion (Scheres & Chen, 2012 ▸). A summary of the data-collection and processing parameters is given in Supplementary Table S2. A schematic overview of the processing is given in Supplementary Fig. S1. The apoferritin map has been deposited in the EMDB (EMD-10533).

## 3. Results   

### 3.1. Droplet sizes in the voltage-assisted spraying approach   

**Figure 1:**
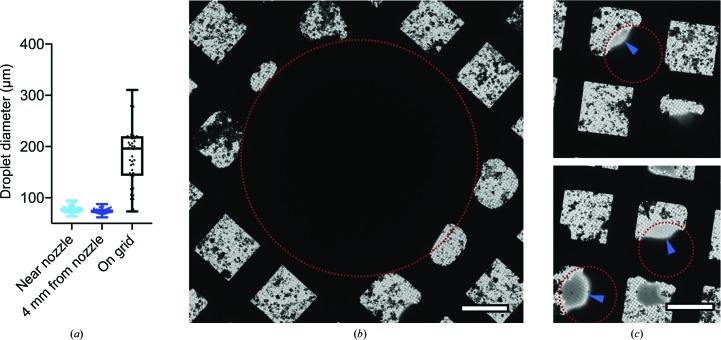
Characterization of droplet spreading after voltage-assisted spraying and freezing. (*a*) The average diameter of spread droplets on the grid is ∼200 µm, which is far in excess of the droplet size generated by the spray. The data are based on 50 observations in-flight and 48 droplets on three grids. (*b*) A large droplet which has not formed a thin film around the periphery of the drop. (*c*) Smaller spread droplets which produce areas suitable for imaging, highlighted with the blue arrows. The red dotted line shows the approximate droplet outline. The scale bar denotes 50 µm.

In an attempt to improve our new rapid mixing and spraying setup (Kontziampasis *et al.*, 2019 ▸), we dissected the voltage-assisted spraying approach to better quantify the relationship of voltage, drop size and speed to droplet spreading on the grid. To generate the spray in the voltage-assisted approach, three main mechanisms are used. (i) Liquid exits the nozzle at a high flow rate, leading to the formation of a liquid jet which eventually breaks up into droplets, (ii) a sheath of N_2_ gas flow is used to aid the breakup of the liquid jet and accelerate the droplets and (iii) an electric potential of 5 kV is applied to the liquid, destabilizing the jet and dispersing the droplets. As previously described, the latter produces a spray consisting mainly of large droplets (∼70 µm diameter) with moderate speed (4–8 m s^−1^; Kontziampasis *et al.*, 2019 ▸). Using the voltage-assisted spraying approach, we successfully produced a number of grids using a variety of samples including apoferritin, thin filaments and ribosomes. With this method, a range of spread droplet sizes was found, with an average diameter of ∼200 µm (Fig. 1 ▸ *a*). Assuming a contact angle of 10–15° (Jain *et al.*, 2012 ▸), spherical droplets of 70 µm in diameter would be expected to spread over a circular area of ∼200 µm in diameter, covering multiple grid squares. The low-magnification ‘Atlas’ views collected for the sprayed grids were consistent with large spread droplets being most abundant on the grids after spraying (Figs. 1 ▸ *a* and 1 ▸ *b*). However, thinning of the ice suitable for data collection was more frequently observed for smaller droplets with a <100 µm spread diameter (Fig. 1 ▸ *c*). As in previous studies, we noticed the presence of background contamination (dark regions on the grid). These are also present when only buffer components are sprayed, suggesting that these are not aggregated protein but ice contamination.

### 3.2. Liquid jets for the deposition of droplets with constant size   

If the thinning of the droplet on the grid is solely dependent on droplet size, then an approach which produces smaller droplets should be better suited for cryo-EM grid preparation. To study the relationship between droplet size and thin-film formation in more detail, we required a method for the reliable production of droplets that were both smaller and more consistent in size. To this end, we adopted the use of Rayleigh jets, where liquid is pushed through an open capillary to form a liquid jet, the diameter of which typically adopts that of the inner diameter (ID) of the capillary. As the jet travels away from the nozzle, it accumulates instabilities (Rayleigh instabilities) and eventually breaks up into droplets. The droplet size after jet breakup is primarily dependent on the jet diameter and therefore on the ID of the nozzle (van Hoeve *et al.*, 2010 ▸; Eggers & Villermaux, 2008 ▸).

**Figure 2:**
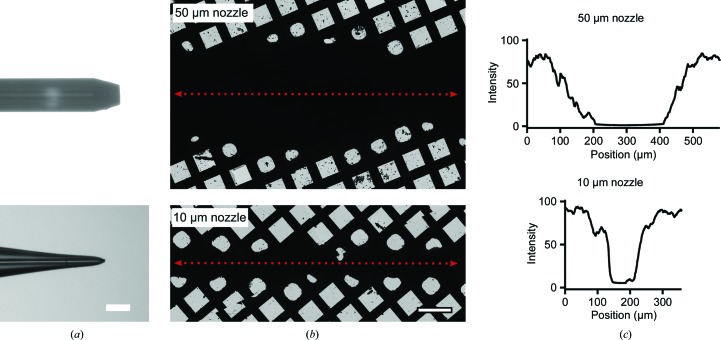
Deposition of droplets from Rayleigh jets. (*a*) Capillaries used for the generation of Rayleigh jets. (*b*) Frozen grids at low magnification in the electron microscope showing a clear strip but no droplet spreading. (*c*) The widths of the ice strips were approximately 200 and 60 µm for the 50 and 10 µm capillaries, respectively. All scale bars correspond to 100 µm.

To investigate the effect of droplet size on the resulting ice quality, we used two different nozzles with 50 or 10 µm ID (Fig. 2 ▸ *a*). Stable jetting could be observed at flow rates of ≥8 and ≥1 µl s^−1^ for the 50 and 10 µm nozzles, respectively. Cryo-EM grids were prepared under these conditions, and low-magnification images of the grids showed a stripe of ice with consistent width across the grid, but no droplet thinning was observed (Fig. 2 ▸ *b*). The nozzle–grid distance was 7 mm, which may provide a sufficient distance for the jet to break up into droplets. Consequently, higher grid speeds led to separated droplets on the surface of the grid for the 10 µm capillary rather than a continuous stripe, but did not promote droplet thinning (Supplementary Fig. S2).

The width of the stripe of thick ice (Fig. 2 ▸ *c*) is consistent with droplet radii of approximately 35 and 10 µm pre-spreading on the grid, assuming a contact angle of 12.5°. This is in agreement with the droplets being larger in diameter than the jet that they originate from (van Hoeve *et al.*, 2010 ▸). Additionally, droplet coalescence occurs on the grids, which will lead to a wider stripe and an overestimated droplet size.

Notably, the droplet sizes (on the frozen grid) stemming from the 10 µm ID capillary are close to the sizes of thinned droplets in voltage-assisted spraying. However, no spreading is observed for these droplets with useable ice at the rims. The difference in spreading implies that the contact angles are different, so droplets covering similar areas on the grid may have different volumes. A possible reason is that droplet speed and spreading upon impact are different, with droplets in the voltage-assisted approach being accelerated through an N_2_ gas flow. The very high backpressure created by the 10 µm ID capillary imposed a limit on the maximum flow rate in our setup and prohibited the use of even smaller capillaries, and therefore limited the attainable droplet speed and size. Blocking of the capillary was also a problem; this was not restricted to a particular protein sample or buffer, but was an inherent drawback of the design. In order to reduce the clogging effect and create smaller or faster droplets, we turned to gas dynamic virtual nozzles (GDVNs; DePonte *et al.*, 2008 ▸).

### 3.3. GDVNs to produce small droplets which form thin films on cryo-EM grids   

**Figure 3:**
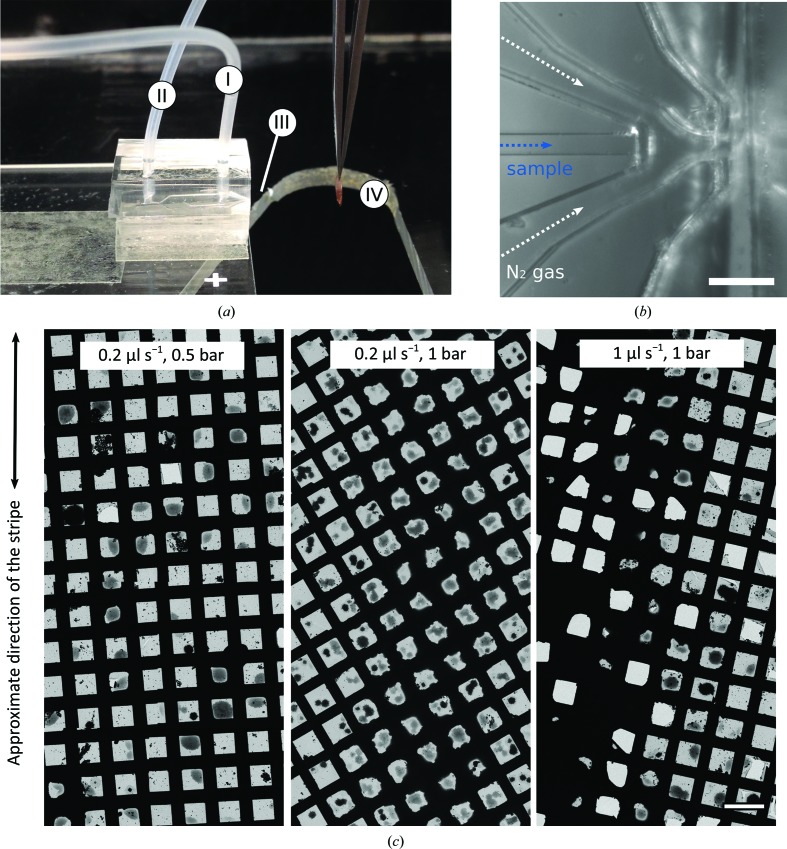
GDVN used for cryo-EM grid preparation. (*a*) The GDVN device fitted within the current setup showing (I) the liquid inlet tubing, (II) the N_2_ gas inlet tubing, (III) the position of the nozzle and (IV) the grid in the target position for spraying. (*b*) Microscopic image of the internal GDVN geometry used in this work with the sample and gas channels labelled. The scale bar denotes 100 µm. (*c*) Typical grids generated with the microfluidic GDVN device under three different conditions. On all three grids, liquid was deposited approximately as a stripe across the grid. The scale bar denotes 100 µm.

The GDVN design was introduced over two decades ago (Gañán-Calvo, 1998 ▸) and has been used for a number of applications, including sample delivery for X-ray free-electron lasers (Chapman *et al.*, 2011 ▸; Wiedorn *et al.*, 2018 ▸). GDVNs are made of two key components: (i) a liquid capillary through which the sample exits at a defined flow rate and (ii) an aperture at a distance from this liquid channel. A pressure drop at this aperture causes gas to flow through it, thereby focusing the liquid into a thin jet and accelerating it. The jet diameter for a given sample is governed by the liquid flow rate and pressure drop in the nozzle, and is typically much smaller than the inner diameter of the liquid inlet (Gañán-Calvo, 1998 ▸). Like the Rayleigh jets described above, the gas-focused liquid jet breaks up into droplets through accumulating instabilities, and the droplet diameter depends on the jet diameter. The GDVN design allows small droplets to be produced with relatively large ID liquid capillaries, which makes this approach less prone to blocking and importantly produces lower backpressure (Trebbin *et al.*, 2014 ▸). The main design used in this work (Figs. 3 ▸ *a* and 3 ▸ *b*) was based on a previous geometry which is capable of producing liquid jets with submicrometre diameters or, when operated at higher gas flows, fine sprays (Trebbin *et al.*, 2014 ▸).

Using the GDVN nozzle to deliver sample onto a moving EM grid, we found a large number of droplets spreading at significantly reduced liquid flow rates (Fig. 3 ▸ *c*). As expected, the droplets are distributed as a stripe on the grid, since they originate from a narrow jet and the distance between the nozzle and grid is low (7–15 mm). At the lowest flow rate tested (0.2 µl s^−1^) and a low applied N_2_ gas pressure (0.5 bar), the deposited liquid volume is sufficiently low to avoid the formation of a thick ice layer. Importantly, the droplets instead spread out and produce areas with ice of appropriate thickness for cryo-EM imaging (left panel in Fig. 3 ▸ *c*). Increasing the N_2_ gas flow leads to a thinner liquid jet and smaller droplets, which lead to a large number of areas with thin ice (middle panel in Fig. 3 ▸ *c*). If the liquid flow rate is increased to ≥1 µl s^−1^ (at 1 bar applied sheath-gas pressure), the amount of liquid becomes too high in the centre of the deposited stripe, resulting in very thick, crystalline ice that is unsuitable for imaging (right panel in Fig. 3 ▸ *c*). Droplets at a distance from the central stripe, however, show spreading and produce areas that are suitable for imaging. This can be accounted for in part by changing the speed at which the grid passes in front of the spray, but in our current setup this can only be altered within a relatively narrow range. At even higher liquid flow rates (≥2 µl s^−1^) we were not able to produce sufficiently small droplets running the GDVN nozzle in jetting mode, resulting in grids similar to that shown in Fig. 2 ▸(*b*) (bottom panel).

### 3.4. From liquid jets to sprays   

**Figure 4:**
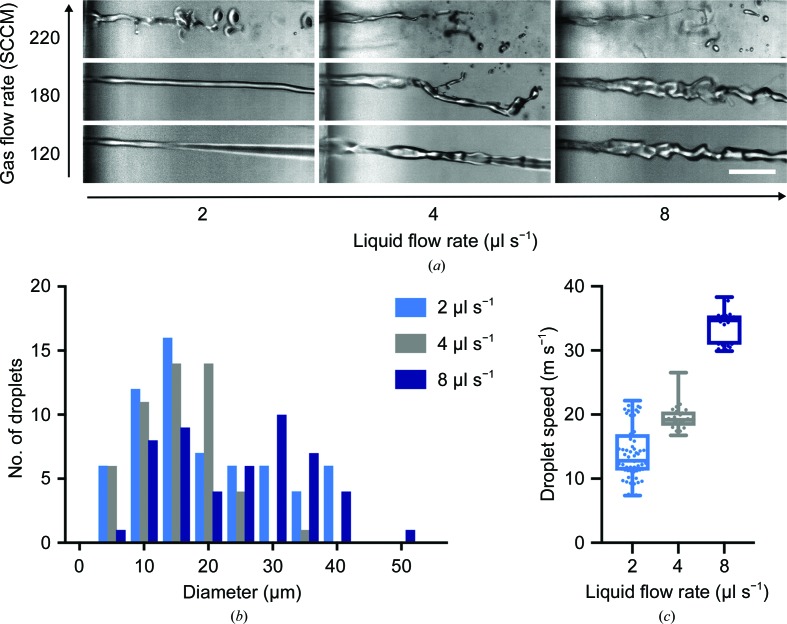
(*a*) High-speed imaging at the GDVN orifice of the jet/spray transition resulting from increasing gas and liquid flow rates. Bursts of 3000 consecutive frames were collected at 200 000 frames per second (fps) with an exposure time of 10 ns (pulsed laser illumination). The scale bar is 100 µm. (*b*) Droplet diameter distribution for a 220 SCCM N_2_ flow. (*c*) Droplet-speed distribution at 220 SCCM.

Using the same GDVN geometry, we explored the use of higher N_2_ flow rates (1–2 bar) at high liquid flow rates (≥2 µl s^−1^). Using high-speed imaging, we found a transition from well defined jets to more chaotic sprays at N_2_ gas-flow rates of ∼200 standard cubic centimetres per minute (SCCM) (approximately 2 bar pressure) for liquid flow rates of up to 8 µl s^−1^ (Fig. 4 ▸ *a*). The formation of these sprays is quite different from the gas-focused jets described earlier. Owing to the high gas and liquid flow rates, high turbulence at the liquid surface leads to faster and more chaotic jet breakup, which occurs at the orifice of the nozzle.

At these increased gas and liquid flow rates, we observed a wider spray cone of smaller and faster droplets covering a larger area on the grid compared with the gas-focused jetting mode. We found a bimodal distribution of droplet sizes of between 5 and 40 µm in diameter (Fig. 4 ▸ *b*) when running at the highest tested liquid flow rate (8 µl s^−1^) and at 220 SCCM N_2_, but this trend was not evident at lower liquid flow rates, where most droplets had a size of <20 µm. The droplet speed depended mostly on the liquid flow rate, with the fastest droplets reaching a speed of 35 m s^−1^ (Fig. 4 ▸ *c*). With our grid-preparation setup, we also found the spraying mode to be a more robust approach than the jetting mode and to tolerate imperfections in the nozzle much more, for example from manufacturing errors or contamination from the sample.

Based on the aforementioned results, these small and high-velocity droplets should be ideally suited for deposition and thinning liquid samples on cryo-EM grids. To verify this, we chose apoferritin as a specimen and prepared cryo-EM grids for data collection operating the GDVN nozzle in spraying mode. We used a 4 µl s^−1^ liquid flow rate and a high N_2_ gas pressure (2 bar); under these conditions the spray consists of very small droplets compared with the voltage-assisted approach (compare Figs. 4 ▸ *b* and 1 ▸ *a*) and the droplets are significantly faster (∼20 m s^−1^ versus ∼6 m s^−1^). Using equine apoferritin as a sample, 690 micrographs were collected from a grid prepared with a time delay of 36 ms between spraying and freezing. The resultant apoferritin structure was determined to 3.5 Å resolution, which is consistent with the resolution achieved using the voltage-assisted approach (Kontziampasis *et al.*, 2019 ▸). The preparation of this grid was reproducible and more efficient compared with the voltage-assisted approach: only 4 µl of sample was used per grid, as the spray from the GDVN nozzle stabilizes more quickly (0.5 s) and was operated at a lower liquid flow rate.

## 4. Discussion   

The grid-preparation stage of single-particle cryo-EM is still an area for development, with problems in grid quality, consistency and interactions with the air–water interface. The traditional blotting method has been highly successful for many samples, but does not come without its limitations, such as sample loss and protein instability for various systems. Alternatively, one can spray or deposit the sample directly onto the grid to eliminate interactions with the filter paper and often reduce the time that the protein resides within a thin film before plunging and freezing. The direct spraying approach for cryo-EM grid preparation (introduced in the early days of cryo-EM; Dubochet *et al.*, 1982 ▸) has not been extensively used, as generating a film that is thin enough for single-particle imaging presents a significant challenge. However, one major advantage of sample spraying is the possibility of introducing a mixing step for time-resolved cryo-EM experiments (Berriman & Unwin, 1994 ▸; Walker *et al.*, 1999 ▸; Chen *et al.*, 2015 ▸).

More recently, there have been a handful of reports on direct spray devices within the literature for cryo-EM. The voltage-assisted approach discussed in this work uses a nozzle where the capillary ends flush with the nozzle or protrudes beyond the gas outlet. A similar design, without the use of high voltage, has been described by Wagenknecht and coworkers (Lu *et al.*, 2014 ▸, 2009 ▸). In that device, the breakup of the liquid stream into droplet occurs outside the injector geometry and is purely driven by the gas stream. In GDVN nozzles under spraying conditions (high liquid and gas flows), the gas and the liquid come into contact within the nozzle. At the nozzle orifice, the expansion of the gas and the fast velocity of both fluids leads to chaotic instabilities, resulting in more efficient atomization and a fine spray. This is similar to the ‘internal-atomization’ sprayer developed by Frank and coworkers, in which atomization occurs within the nozzle, allowing a degree of control over the resulting droplet size (Feng *et al.*, 2017 ▸). Additionally, GDVNs can be used in jetting mode at low liquid and gas flow rates, where the liquid stream is focused to low diameters by a coaxial gas flow. Breakup of the jet (outside the device) then produces a narrow range of droplet sizes downstream.

In this work, we wanted to provide a better understanding of the droplet behaviour on a commercially available EM grid that had not been modified other than by glow-discharge treatment, using the direct spraying approach. We observed broken areas on the grid to varying extents (Figs. 3 ▸ and 5 ▸), depending on the grid foil and mesh type and spray conditions (liquid and gas flow). Using 300 mesh grids and R1.2/1.3 foil, however, the number of intact grid squares is high and sufficient for the collection of >1000 micrographs. Our initial results suggest that droplet size is an important parameter in forming a suitable film on a fast-moving cryo-EM grid but may not be the sole determinant. We hypothesize that droplet speed could play an important role in overcoming the challenge of appropriate surface wetting in forming a very thin film. The GDVNs presented in this work can produce small and high-velocity droplets over a wide range of liquid flow rates, operating in jetting (low liquid flow rates of <2 µl s^−1^, medium gas pressures of ∼1 bar) or spraying mode (high liquid flow rates of ≥2 µl s^−1^, high gas pressures of ≥2 bar). The jetting mode allows grid preparation with much lower sample consumption per grid than previous spraying approaches (∼1 µl versus tens of microlitres), but still suffers from the large dead volume in our setup (∼30 µl). However, the dead volume of this setup can be lowered significantly in future iterations by reducing the tubing ID and length of the liquid lines. The spraying approach allows higher liquid flow rates which might be needed upstream, in a mixing unit for example, or for generating faster droplets if the grid is moved at very high velocity. Our apoferritin reconstruction shows that these droplets are equally as suitable for cryo-EM data collection as those produced by the voltage-assisted approach. The benefits of the GDVN gas-flow focusing geometry could be combined with high voltage in the future to offer an additional parameter to control droplet size and behaviour in a voltage-assisted GDVN spraying device (Ganán-Calvo, 2007 ▸).

Additionally, other solutions are available to control droplet spreading, one of which is the recent development of self-wicking grids by Wei *et al.* (2018 ▸), which have proven to be successful not only for piezo-dispensing but also for use with ultrasonic sprayers (Rubinstein *et al.*, 2019 ▸). This grid type is expected to also improve droplet spreading in the setup described in this work and can offer the potential for far more areas of thinner ice. We look forward to seeing how we can further increase the quality and consistency of the ice thickness through self-wicking grids in combination with changes in droplet size, droplet velocity and voltage, giving a choice of variables to modulate droplet behaviour depending on the experimental setup and sample properties.

The aim of the setup that we have designed is to study the rapid mixing of proteins and substrates in order to, for example, trap distinct states, rapidly change the pH or solute concentration and better understand protein-complex mechanisms (Levantino *et al.*, 2015 ▸). Currently, the minimum time delay between sample exiting the nozzle and vitrification is ∼5 ms, but one of the main limitations for going faster is the ice thickness at faster plunge speeds. We anticipate that optimization of the droplet size and velocity together with modification of the grid surface properties will help to achieve shorter time delays and ultimately allow grid preparation in a sub-millisecond timeframe. Although sub-4 Å resolution is sufficient to address many biological questions, by further understanding droplet thinning on the grid we aim to achieve sub-3 Å spatial resolution (through improved ice quality) in combination with faster grid preparation and shorter time delays. There is still work to be performed towards this goal but, as with many developments in the cryo-EM field, the future looks promising.

## Supplementary Material

EMDB reference: apoferritin, EMD-10533

Supplementary Figures. DOI: 10.1107/S2059798320002958/ij5002sup1.pdf

## Figures and Tables

**Figure 5:**
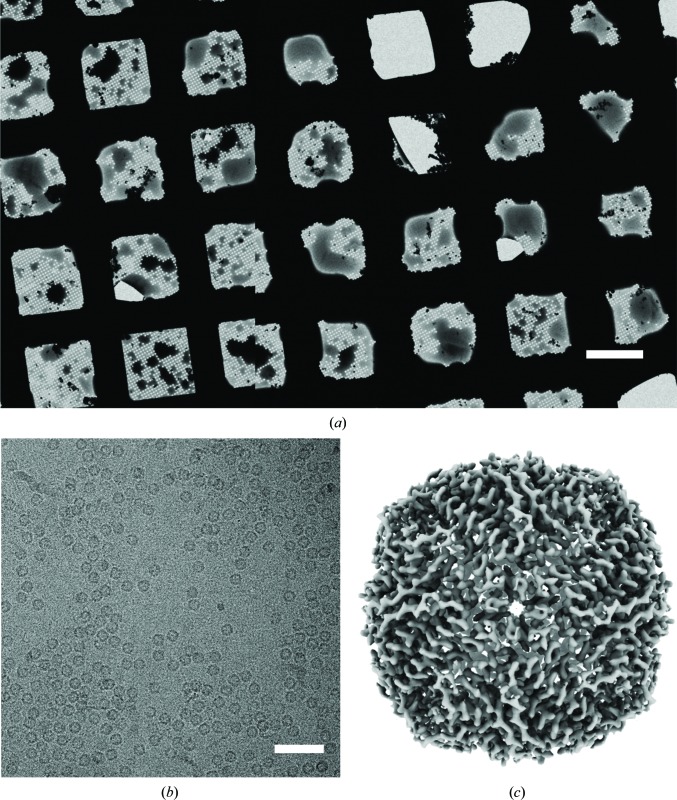
(*a*) Low-magnification cryo-electron micrograph of a grid prepared using the microfluidic GDVN device in spraying mode (liquid at 4 µl s^−1^, gas at 2 bar). The scale bar corresponds to 50 µm. (*b*) Representative high-magnification image of an area with thin ice used for data collection (the scale bar corresponds to 50 nm). (*c*) Single-particle reconstruction of apoferritin to 3.5 Å resolution with data collected from a single grid made using the GDVN nozzle under spraying conditions (liquid at 4 µl s^−1^, gas at 2 bar).

## References

[bb1] Armstrong, M., Han, B.-G., Gomez, S., Turner, J., Fletcher, D. A. & Glaeser, R. M. (2019). *bioRxiv*, 791285.

[bb2] Arnold, S. A., Albiez, S., Bieri, A., Syntychaki, A., Adaixo, R., McLeod, R. A., Goldie, K. N., Stahlberg, H. & Braun, T. (2017). *J. Struct. Biol.* **197**, 220–226. 10.1016/j.jsb.2016.11.002 27864160

[bb3] Ashtiani, D., Venugopal, H., Belousoff, M., Spicer, B., Mak, J., Neild, A. & de Marco, A. (2018). *J. Struct. Biol.* **203**, 94–101. 10.1016/j.jsb.2018.03.012 29630922

[bb4] Berriman, J. & Unwin, N. (1994). *Ultramicroscopy*, **56**, 241–252. 10.1016/0304-3991(94)90012-4 7831735

[bb5] Callaway, E. (2015). *Nature*, **525**, 172–174. 10.1038/525172a 26354465

[bb6] Chapman, H. N., Fromme, P., Barty, A., White, T. A., Kirian, R. A., Aquila, A., Hunter, M. S., Schulz, J., DePonte, D. P., Weierstall, U., Doak, R. B., Maia, F. R. N. C., Martin, A. V., Schlichting, I., Lomb, L., Coppola, N., Shoeman, R. L., Epp, S. W., Hartmann, R., Rolles, D., Rudenko, A., Foucar, L., Kimmel, N., Weidenspointner, G., Holl, P., Liang, M., Barthelmess, M., Caleman, C., Boutet, S., Bogan, M. J., Krzywinski, J., Bostedt, C., Bajt, S., Gumprecht, L., Rudek, B., Erk, B., Schmidt, C., Hömke, A., Reich, C., Pietschner, D., Strüder, L., Hauser, G., Gorke, H., Ullrich, J., Herrmann, S., Schaller, G., Schopper, F., Soltau, H., Kühnel, K., Messerschmidt, M., Bozek, J. D., Hau-Riege, S. P., Frank, M., Hampton, C. Y., Sierra, R. G., Starodub, D., Williams, G. J., Hajdu, J., Timneanu, N., Seibert, M. M., Andreasson, J., Rocker, A., Jönsson, O., Svenda, M., Stern, S., Nass, K., Andritschke, R., Schröter, C., Krasniqi, F., Bott, M., Schmidt, K. E., Wang, X., Grotjohann, I., Holton, J. M., Barends, T. R. M., Neutze, R., Marchesini, S., Fromme, R., Schorb, S., Rupp, D., Adolph, M., Gorkhover, T., Andersson, I., Hirsemann, H., Potdevin, G., Graafsma, H., Nilsson, B. & Spence, J. C. H. (2011). *Nature*, **470**, 73–77.

[bb7] Chen, B., Kaledhonkar, S., Sun, M., Shen, B., Lu, Z., Barnard, D., Lu, T.-M., Gonzalez, R. L. Jr & Frank, J. (2015). *Structure*, **23**, 1097–1105. 10.1016/j.str.2015.04.007 PMC4456197 26004440

[bb8] Cheng, Y. (2018). *Science*, **361**, 876–880. 10.1126/science.aat4346 PMC6460916 30166484

[bb9] Dandey, V. P., Wei, H., Zhang, Z., Tan, Y. Z., Acharya, P., Eng, E. T., Rice, W. J., Kahn, P. A., Potter, C. S. & Carragher, B. (2018). *J. Struct. Biol.* **202**, 161–169. 10.1016/j.jsb.2018.01.002 PMC6317895 29366716

[bb40] DePonte, J., Weierstall, U., Schmidt, K., Warner, J., Starodub, D., Spence, J. C. H. & Doak, R. B. (2008). *J. Phys. D Appl. Phys.* **41**, 195505.

[bb10] D’Imprima, E., Floris, D., Joppe, M., Sánchez, R., Grininger, M. & Kühlbrandt, W. (2019). *eLife*, **8**, e42747. 10.7554/eLife.42747 PMC6443348 30932812

[bb11] Dubochet, J., Adrian, M., Lepault, J. & McDowall, A. (1985). *Trends Biochem. Sci.* **10**, 143–146.

[bb12] Dubochet, J., Lepault, J., Freeman, R., Berriman, J. & Homo, J. C. (1982). *J. Microsc.* **128**, 219–237.

[bb13] Eggers, J. & Villermaux, E. (2008). *Rep. Prog. Phys.* **71**, 036601.

[bb14] Feng, X., Fu, Z., Kaledhonkar, S., Jia, Y., Shah, B., Jin, A., Liu, Z., Sun, M., Chen, B., Grassucci, R. A., Ren, Y., Jiang, H., Frank, J. & Lin, Q. (2017). *Structure*, **25**, 663–670. 10.1016/j.str.2017.02.005 PMC5382802 28286002

[bb15] Gañán-Calvo, A. M. (1998). *Phys. Rev. Lett.* **80**, 285–288.

[bb16] Ganán-Calvo, A. M. (2007). *Phys. Rev. Lett.* **98**, 134503. 10.1103/PhysRevLett.98.134503 17501206

[bb17] Hoeve, W. van, Gekle, S., Snoeijer, J. H., Versluis, M., Brenner, M. P. & Lohse, D. (2010). *Phys. Fluids*, **22**, 122003.

[bb18] Jain, T., Sheehan, P., Crum, J., Carragher, B. & Potter, C. S. (2012). *J. Struct. Biol.* **179**, 68–75. 10.1016/j.jsb.2012.04.020 PMC3378829 22569522

[bb19] Kaledhonkar, S., Fu, Z., Caban, K., Li, W., Chen, B., Sun, M., Gonzalez, R. L. & Frank, J. (2019). *Nature*, **570**, 400–404. 10.1038/s41586-019-1249-5 PMC7060745 31108498

[bb20] Kontziampasis, D., Klebl, D. P., Iadanza, M. G., Scarff, C. A., Kopf, F., Sobott, F., Monteiro, D. C. F., Trebbin, M., Muench, S. P. & White, H. D. (2019). *IUCrJ*, **6**, 1024–1031. 10.1107/S2052252519011345 PMC6830222 31709058

[bb41] Levantino, M., Yorke, B. A., Monteiro, D. C. F., Cammarata, M. & Pearson, A. R. (2015). *Curr. Opin. Struct. Biol.* **35**, 41–48. 10.1016/j.sbi.2015.07.017 26342489

[bb21] Lu, Z., Barnard, D., Shaikh, T. R., Meng, X., Mannella, C. A., Yassin, A. S., Agrawal, R. K., Wagenknecht, T. & Lu, T.-M. (2014). *J. Micromech. Microeng.* **24**, 115001. 10.1088/0960-1317/24/11/115001 PMC4266110 25530679

[bb22] Lu, Z., Shaikh, T. R., Barnard, D., Meng, X., Mohamed, H., Yassin, A., Mannella, C. A., Agrawal, R. K., Lu, T.-M. & Wagenknecht, T. (2009). *J. Struct. Biol.* **168**, 388–395. 10.1016/j.jsb.2009.08.004 PMC2783284 19683579

[bb23] Noble, A. J., Dandey, V. P., Wei, H., Brasch, J., Chase, J., Acharya, P., Tan, Y. Z., Zhang, Z., Kim, L. Y. & Scapin, G. (2018). *eLife*, **7**, e34257. 10.7554/eLife.34257 PMC5999397 29809143

[bb24] Ravelli, R. B. G., Nijpels, F. J. T., Henderikx, R. J. M., Weissenberger, G., Thewessem, S., Gijsbers, A., Beulen, B. W. A. M. M., Lopez-Iglesias, C. & Peters, P. (2019). *bioRxiv*, 651208. 10.1038/s41467-020-16392-5 PMC7244535 32444637

[bb25] Rubinstein, J. L., Guo, H., Ripstein, Z. A., Haydaroglu, A., Au, A., Yip, C. M., Di Trani, J. M., Benlekbir, S. & Kwok, T. (2019). *Acta Cryst.* D**75**, 1063–1070. 10.1107/S2059798319014372 PMC6889916 31793900

[bb26] Scheres, S. H. W. & Chen, S. (2012). *Nat. Methods*, **9**, 853–854. 10.1038/nmeth.2115 PMC4912033 22842542

[bb27] Trebbin, M., Krüger, K., DePonte, D., Roth, S. V., Chapman, H. N. & Förster, S. (2014). *Lab Chip*, **14**, 1733–1745. 10.1039/c3lc51363g 24671443

[bb28] Wagner, T., Merino, F., Stabrin, M., Moriya, T., Antoni, C., Apelbaum, A., Hagel, P., Sitsel, O., Raisch, T., Prumbaum, D., Quentin, D., Roderer, D., Tacke, S., Siebolds, B., Schubert, E., Shaikh, T. R., Lill, P., Gatsogiannis, C. & Raunser, S. (2019). *Commun. Biol.* **2**, 218. 10.1038/s42003-019-0437-z PMC6584505 31240256

[bb29] Walker, M., White, H., Belknap, B. & Trinick, J. (1994). *Biophys. J.* **66**, 1563–1572. 10.1016/S0006-3495(94)80948-8 PMC1275876 8061205

[bb30] Walker, M., Zhang, X.-Z., Jiang, W., Trinick, J. & White, H. D. (1999). *Proc. Natl Acad. Sci. USA*, **96**, 465–470. 10.1073/pnas.96.2.465 PMC15159 9892656

[bb31] Wei, H., Dandey, V. P., Zhang, Z., Raczkowski, A., Rice, W. J., Carragher, B. & Potter, C. S. (2018). *J. Struct. Biol.* **202**, 170–174. 10.1016/j.jsb.2018.01.001 PMC5864531 29317278

[bb32] Wiedorn, M. O., Oberthür, D., Bean, R., Schubert, R., Werner, N., Abbey, B., Aepfelbacher, M., Adriano, L., Allahgholi, A., Al-Qudami, N., Andreasson, J., Aplin, S., Awel, S., Ayyer, K., Bajt, S., Barák, I., Bari, S., Bielecki, J., Botha, S., Boukhelef, D., Brehm, W., Brockhauser, S., Cheviakov, I., Coleman, M. A., Cruz-Mazo, F., Danilevski, C., Darmanin, C., Doak, R. B., Domaracky, M., Dörner, K., Du, Y., Fangohr, H., Fleckenstein, H., Frank, M., Fromme, P., Gañán-Calvo, A. M., Gevorkov, Y., Giewekemeyer, K., Ginn, H. M., Graafsma, H., Graceffa, R., Greiffenberg, D., Gumprecht, L., Göttlicher, P., Hajdu, J., Hauf, S., Heymann, M., Holmes, S., Horke, D. A., Hunter, M. S., Imlau, S., Kaukher, A., Kim, Y., Klyuev, A., Knoška, J., Kobe, B., Kuhn, M., Kupitz, C., Küpper, J., Lahey-Rudolph, J. M., Laurus, T., Le Cong, K., Letrun, R., Xavier, P. L., Maia, L., Maia, F. R. N. C., Mariani, V., Messerschmidt, M., Metz, M., Mezza, D., Michelat, T., Mills, G., Monteiro, D. C. F., Morgan, A., Mühlig, K., Munke, A., Münnich, A., Nette, J., Nugent, K. A., Nuguid, T., Orville, A. M., Pandey, S., Pena, G., Villanueva-Perez, P., Poehlsen, J., Previtali, G., Redecke, L., Riekehr, W. M., Rohde, H., Round, A., Safenreiter, T., Sarrou, I., Sato, T., Schmidt, M., Schmitt, B., Schönherr, R., Schulz, J., Sellberg, J. A., Seibert, M. M., Seuring, C., Shelby, M. L., Shoeman, R. L., Sikorski, M., Silenzi, A., Stan, C. A., Shi, X., Stern, S., Sztuk-Dambietz, J., Szuba, J., Tolstikova, A., Trebbin, M., Trunk, U., Vagovic, P., Ve, T., Weinhausen, B., White, T. A., Wrona, K., Xu, C., Yefanov, O., Zatsepin, N., Zhang, J., Perbandt, M., Mancuso, A. P., Betzel, C., Chapman, H. & Barty, A. (2018). *Nat. Commun.* **9**, 4025.

[bb33] Zhang, K. (2016). *J. Struct. Biol.* **193**, 1–12. 10.1016/j.jsb.2015.11.003 PMC4711343 26592709

[bb34] Zheng, S. Q., Palovcak, E., Armache, J.-P., Verba, K. A., Cheng, Y. & Agard, D. A. (2017). *Nat. Methods*, **14**, 331–332. 10.1038/nmeth.4193 PMC5494038 28250466

[bb35] Zivanov, J., Nakane, T., Forsberg, B. O., Kimanius, D., Hagen, W. J., Lindahl, E. & Scheres, S. H. W. (2018). *eLife*, **7**, e42166. 10.7554/eLife.42166 PMC6250425 30412051

[bb36] Zivanov, J., Nakane, T. & Scheres, S. H. W. (2019). *IUCrJ*, **6**, 5–17. 10.1107/S205225251801463X PMC6327179 30713699

