# Supplementary material for: Sample deposition onto cryo-EM grids: from sprays to jets and back
Source: Acta Crystallogr D Struct Biol. 2020 Mar 25;76(Pt 4):340–9. doi: 10.1107/S2059798320002958 (PMC7137104; doi:10.1107/S2059798320002958)
Supplement: Supplementary file 1 [file d-76-00340-sup1.pdf]

STRUCTURAL BIOLOGY

Volume 76 (2020)

Supporting information for article:

Sample deposition onto cryo-EM grids: from sprays to jets and back

David P. Klebl, Diana C. F. Monteiro, Dimitrios Kontziampasis, Florian Kopf, Frank Sobott, Howard D. White, Martin Trebbin and Stephen P. Muench

Table S1 Design parameters for the GDVN devices used in this work.

Liquid inlet ID	30 μm
Nozzle opening	30 μm
Inlet to nozzle distance	125 μm

Table S2 Data collection and processing parameters for the apoferritin dataset.

Data collection	
Magnification	130,000 x
Voltage [kV]	300
Pixel size [Å]	1.07
Electron Dose [e^-/Å^2]	60
Defocus range [μm]	0.8 - 2.8
Number of micrographs	690
**Processing**	
Initial particle number	70,425
Final particle number	9,633
Symmetry	O
Global resolution (FSC = 0.143) [Å]	3.5

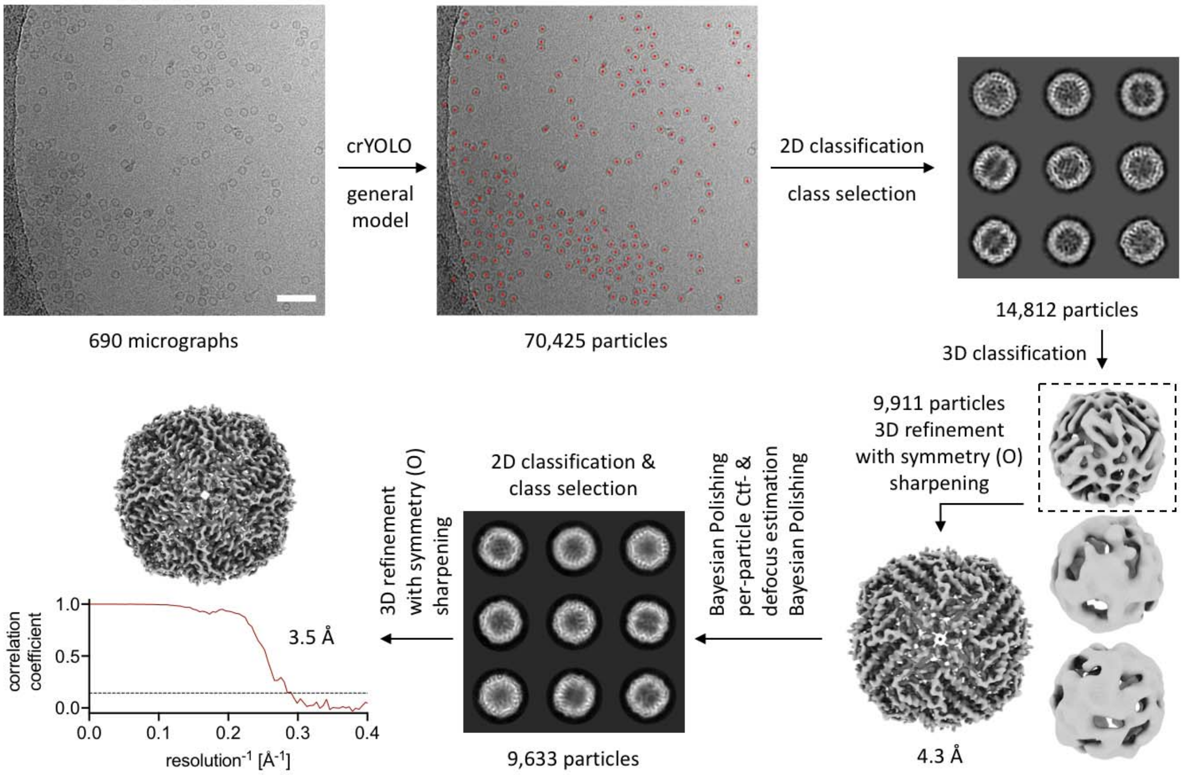

Figure S1 Processing flowchart for apoferritin dataset. The scale bar in the micrograph corresponds to 50 nm.

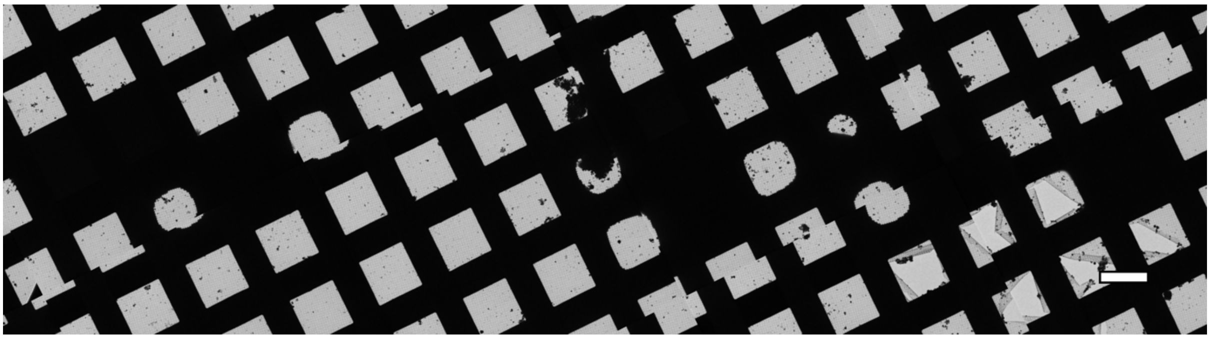

Figure S2 Sample deposition using a Rayleigh jet emitted from the 10 µm capillary at 1.4 m/s grid speed. Droplets show little or no coalescence on the grid and do not spread. Scale bar denotes 50 µm.
